# Supplementary material for: Nicotinamide N-Methyl Transferase (NNMT) Sustains Innate Sensitivity to NAMPT Inhibition in YAP-dependent Stem-like/Mesenchymal Prostate Cancer
Source: Int J Biol Sci. 2026 Jan 1;22(3):1126–41. doi: 10.7150/ijbs.120532 (PMC12836488; doi:10.7150/ijbs.120532)
Supplement: Supplementary file 1 — Supplementary figures and material 1 and 2. [file ijbsv22p1126s1.pdf]

Nicotinamide N-methyl transferase (NNMT) sustains innate sensitivity to NAMPT inhibition in YAP-dependent stem-like/ mesenchymal prostate cancer

Ágata Sofia Assunção Carreira[1,#], Marianna Ciuffreda[2], Nathakan Thongon[1], Charles M. Haughey[3], Adam Pickard[3], Sharon L. Eddie[3], Rebecca E. Steele[3], Elena Cerri[1], Romina Belli[4], Daniele Peroni[4], Elisa Facen[1], Irene Caffa[5,6], Moustafa Ghanem[5], Alessio Nencioni[5,6], Andrea Lunardi[1], Toma Tebaldi[2], Ian G. Mills[3,7], Alessandro Provenzani,[1,*]

SUPPLEMENTARY FIGURES

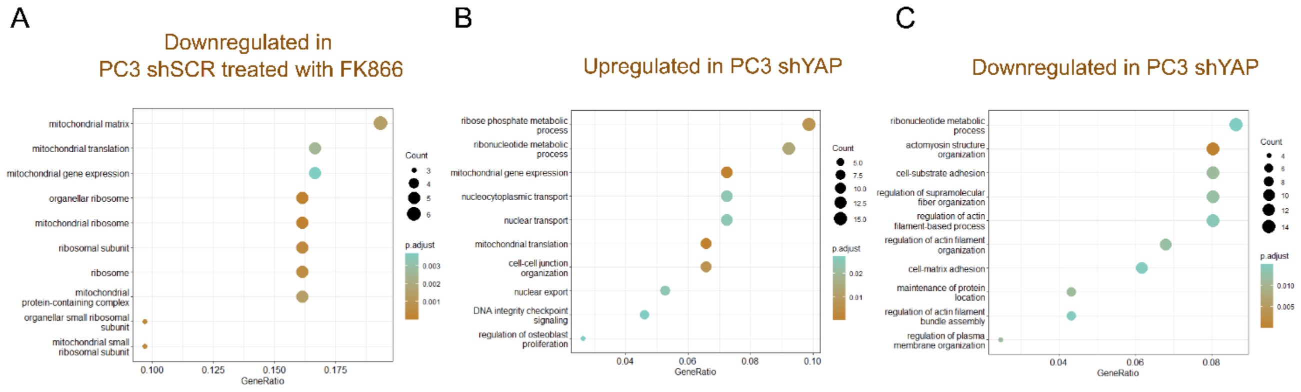

Supplementary Figure 1: Gene set enrichment analysis (GSEA) of proteomics data. **(A)** GSEA of downregulated proteins in PC3 shSCR cells treated with FK866 (5nM) for 48 hours **(B)** GSEA of proteins upregulated or (C) downregulated in PC3 shYAP, compared with the scramble condition (PC3 shSCR). Significantly modulated proteins presented a p-value above 0,05, calculated from three independent biological replicates.

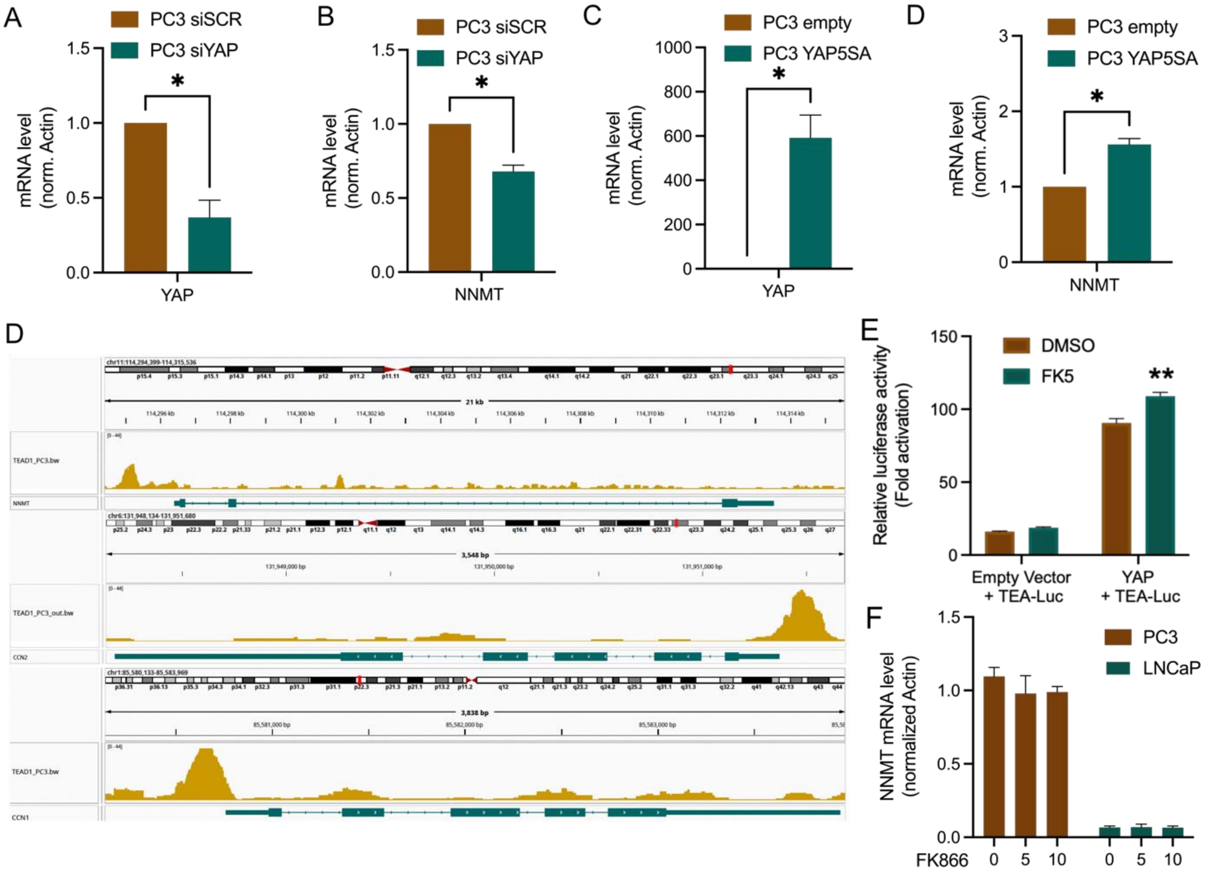

Supplementary Figure 2: mRNA levels of (A), (C) *YAP* and (B), (D) *NNMT* in PC3 cells transfected either with siRNA targeting YAP or with plasmids overexpressing constitutively active YAP (YAP5SA) for 48 hours. Actin was used as the housekeeping gene. (D) ChIP-seq evaluation of TEAD1 binding sites in the promoter region of *NNMT* locus in PC3 cells. (E) Luciferase signal in PC3 cells transfected with either pRGFPN1 (empty vector) or YAP, as well as with the firefly TEA-Luc reporter (8xGTIIC-Luc reporter) and with the Renilla luciferase. Cells were treated with 5 nM of FK866 (FK5) or DMSO for 48 hours. Firefly luciferase signals were normalized to the ones of Renilla luciferase (F) *NNMT* mRNA levels in PC3 and LNCaP cells treated with 5 or 10 nM of FK866 for 48 hours. Actin was used as the housekeeping gene. Student t-test was used to calculate statistical significance (ns- not significant, *P < 0.05, *P<0.01) between control and experimental conditions.

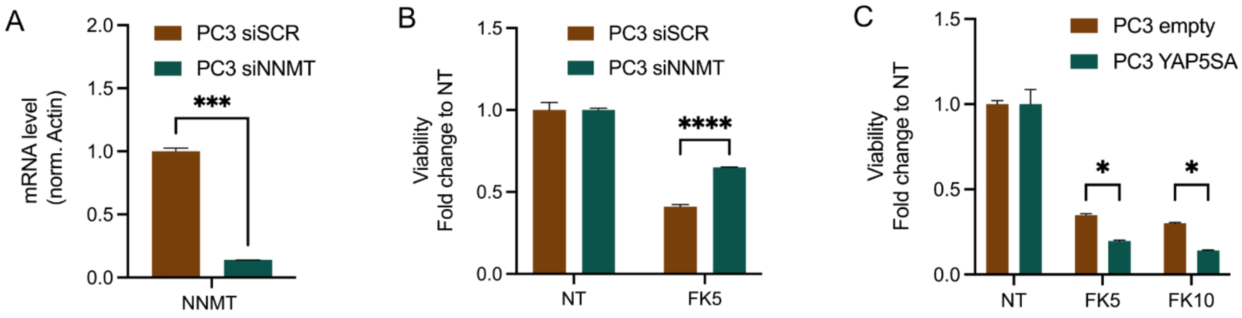

Supplementary Figure 3: (A) mRNA level of NNMT in PC3 cells transiently transfected with siRNAs targeting *NNMT* for 48 hours. siSCR was used as a control of the transfection. Actin was used as a housekeeping gene. **(B)** Viability assay of PC3 cells treated with FK866 at 5 nM (FK5) concentration, for 48 hours, after 24 hours of *NNMT* silencing with siNNMT. **(C)** Viability assay of PC3 cells treated with FK866 at 5 (FK5) or 10 nM (FK10) of concentration, for 48 hours, after 24 hours of transfection with a constitutively active YAP plasmid (YAP5SA) or the empty control (empty). Repeated measures one-way ANOVA was used to calculate statistical significance (ns- not significant, *P < 0.05, **P < 0.01, ***P < 0.001, ****P<0.0001) between control and experimental conditions.

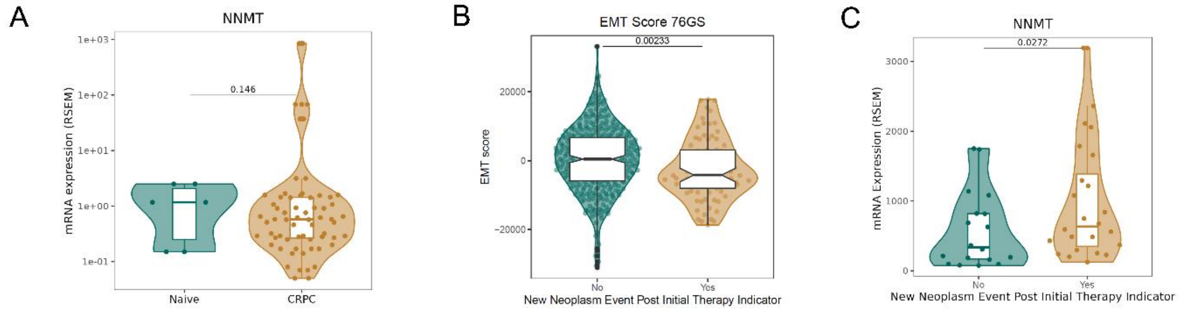

Supplementary Figure 4: (A) NNMT expression levels of PDX samples between naive and CRPC conditions. (**B-C**) Violin plots displaying EMT scores and NNMT mRNA changes, between prostate cancer patients displaying or not a new neoplastic event after treatment. The score was calculated using the 76GS scoring method. Wilcoxon rank-sum test was used to calculate statistical significance across the different conditions.

Supplementary Material 1: list of primers

Gene	Primers (5'-3')
E-cadherin (CDH1)	FW: ACACCCGGGACAACGTTTA
	RV: TGTGCAGCTGGCTCAAGT
N-cadherin (CDH2)	FW: CGGTTTCATTTGAGGGCACA
	RV: TTGGAGCCTGAGACACGATT
Vimentin	FW: GAGAACTTTGCCGTTGAAGC
	RV: GCTTCCTGTAGGTGGCAATC
Snail1	FW: AGTGGTTCTTCTGCGCTACT
	RV: GTAGGGCTGCTGGAAGGTAA
ZEB1	FW: GAAAATGAGCAAAACCATGATCCTA
	RV: CAGGTGCCTCAGGAAAAATGA
NAMPT	FW: TTATGGAACGAAAGATCCTG
	RV: CAAAAGCATCTTTTTCATGGTC
CTGF	FW: CCAATGACAACGCCTCCTG
	RV: TGGTGCAGCCAGAAAGCTC
CYR61	FW: AGCCTCGCATCCTATACAACC
	RV: TTCTTTCACAAGGCGGCACTC
CHOP	FW: AGAACCAGGAAACGGAAACAGA
	RV: TCTCCTTCATGCGCTGCTTT
NNMT	FW: GTTTGGTTCTAGGCACTCTGCAG
	RV: AGAGCCGATGTCAATCAGCAGG
CK8	FW: CGGGGGATCCAACACTTTCA
	RV: GCTTCCCATCTCGGGTTTCA
CK18	FW: CCAGATTGCCAGCTCTGGAT
	RV: ATGTCCGCCATGATCTTGCT
CK5	FW: CAGAGCTGAGGAACATGCAG
	RV: CACAAACTCATTCTCAGCCG
CK14	FW: GGCCCACTGAGATCAAAGAC
	RV: GGCCCACTGAGATCAAAGAC
Twist	FW: GGCCCACTGAGATCAAAGAC
	RV: GGCCCACTGAGATCAAAGAC
Slug	FW: GGCCCACTGAGATCAAAGAC
	RV: GGCCCACTGAGATCAAAGAC
Snail1 (Figure 4)	FW: GGCCCACTGAGATCAAAGAC
	RV: GGCCCACTGAGATCAAAGAC
E-cadherin (CDH1) - Figure 4	FW: GGCCCACTGAGATCAAAGAC
	RV: GGCCCACTGAGATCAAAGAC
N-cadherin (CDH2) - Figure 4	FW: GGCCCACTGAGATCAAAGAC
	RV: GGCCCACTGAGATCAAAGAC
RPLP0	FW: CATTCTCGCTTCCTGGAG
	RV: CTTGACCTTTTCAGCAAGTGG
Beta-actin	FW: AGTGTGACGTTGACATCCGT
	RV: CTTGCTGATCCACATCTGCT

Supplementary Material 2: list of antibodies

Antibodies	Company	Codice
β-actin	Cell Signaling	4970S
GAPDH	Cell signalling	5174S
NNMT	Origene	TA502624
